# Supplementary material for: COVID-19 Outcomes in Patients with Hematologic Malignancies in the Era of COVID-19 Vaccination and the Omicron Variant
Source: Cancers (Basel). 2024 Jan 16;16(2):379. doi: 10.3390/cancers16020379 (PMC10814951; doi:10.3390/cancers16020379)
Supplement: Supplementary file 1 [file cancers-16-00379-s001.zip › cancers-2744585-supplementary.pdf]

Supplementary Materials: COVID-19 Outcomes in Patients with Hematologic Malignancies in the Era of COVID-19 Vaccination and the Omicron Variant

Joaquín Martínez-López, Javier de la Cruz, Rodrigo Gil-Manso, Víctor Jiménez Yuste, José María Aspa-Cilleruelo, Cristian Escolano Escobar, Javier López-Jiménez, Rafael Duarte, Cristina Jacome Yerovi, José-Ángel Hernández-Rivas, Regina Herráez, Keina Quiroz-Cervantes, Rosalía Bustelos-Rodriguez, Celina Benavente, Pilar Martínez Barranco, Mariana Bastos Oteiro, Adrián Alegre, Jaime Pérez-Oteyza, Elena Ruiz, Eriel Alexis Marcheco-Pupo, Ángel Cedillo, Teresa de Soto Álvarez, Patricia García Ramirez, Rosalía Alonso Trillo, Pilar Herrera, María Luisa Bengochea Casado, Andrés Arroyo Barea, Jose Manuel Martin De Bustamante, Javier Ortiz, María Calbacho Robles and Julio García-Suárez on behalf of the Asociación Madrileña de Hematología y Hemoterapia (AMHH)

Table S1. Baseline characteristics of, and therapy received by, patients with hematologic malignancies according to COVID-19 clinical severity, and effect of characteristics on odds of having severe/critical disease.

	**Patients with COVID-19 severity data, N = 1781**			**Adjusted OR***	
	**All, N = 1781 n (%)**	**Mild / Moderate, n = 1020 n (% of 'all')**	**Severe / Critical, n = 761 n (% of 'all')**	**OR (95% CI)**	***P*-value**
**Age, y, n (%)**	**n = 1780**	**n = 1019**	**n = 761**		
<60	499 (28.0)	372/499 (74.5)	127/499 (25.5)	Reference	
60-70	374 (21.0)	222/374 (59.4)	152/374 (40.6)	1.62 (1.21-2.19)	**.001**
70-80	540 (30.3)	269/540 (49.8)	271/540 (50.2)	2.16 (1.64-2.87)	**<.001**
>80	367 (20.6)	156/367 (42.5)	211/367 (57.5)	2.64 (1.93-3.62)	**<.001**
**Sex, n (%)**	**n = 1766**	**n = 1012**	**n = 754**		
Male	1016 (57.5)	550/1016 (54.1)	466/1016 (45.9)	Reference	
Female	750 (42.5)	462/750 (61.6)	288/750 (38.4)	0.79 (0.64-0.96)	**.021**
**Comorbidities, n (%)**	**n = 1781**	**n = 1020**	**n = 761**		
0	694 (39.0)	505/694 (72.8)	189/694 (27.2)	Reference	
1	635 (35.7)	323/635 (50.9)	312/635 (49.1)	2.02 (1.59-2.57)	**<.001**
>1	452 (25.4)	192/452 (42.5)	260/452 (57.5)	2.44 (1.85-3.21)	**<.001**
**Time period (by variant), n (%)**	**n = 1781**	**n = 1020**	**n = 761**		
Pre-Omicron	1246 (70.0)	579/1246 (46.5)	667/1246 (53.5)	Reference	
Omicron	535 (30.0)	441/535 (82.4)	94/535 (17.6)	0.21 (0.16-0.27)	**<.001**
Omicron BA.1 or BA.2	321/535 (60.0)	253/321 (78.8)	68/321 (21.2)	0.28 (0.20-0.37)	**<.001**
Omicron BA.4 or BA.5	214/535 (40.0)	188/214 (87.9)	26/214 (12.1)	0.13 (0.08-0.19)	**<.001**
**Hematologic malignancy, n (%)**	**n = 1780**	**n = 1019**	**n = 761**		
Lymphoid malignancy	1326 (74.5)	777/1326 (58.6)	549/1326 (41.4)	Reference (vs myeloid malignancy category)	
NHL	543/1326 (41.0)	327/543 (60.2)	216/543 (39.8)	Reference (vs individual malignancies)	
ALL	53/1326 (4.0)	35/53 (66.0)	18/53 (34.0)	1.62 (0.85-3.02)	.1
CLL	241/1326 (18.2)	105/241 (43.6)	136/241 (56.4)	1.64 (1.19-2.27)	**.003**
HL	74/1326 (5.6)	50/74 (67.6)	24/74 (32.4)	0.90 (0.51-1.55)	.7
MM	415/1326 (31.3)	260/415 (62.7)	155/415 (37.3)	0.76 (0.58-1.01)	.06
Myeloid malignancy	454 (25.5)	242/454 (53.3)	212/454 (46.7)	1.07 (0.85-1.34)	.6
AML	147/454 (32.4)	79/147 (53.7)	68/147 (46.3)	1.44 (0.98-2.13)	.06
CML	42/454 (9.3)	29/42 (69.0)	13/42 (31.0)	0.71 (0.34-1.42)	.3
MDS	141/454 (31.1)	64/141 (45.4)	77/141 (54.6)	1.12 (0.75-1.67)	.6

MPN	124/454 (27.3)	70/124 (56.5)	54/124 (43.5)	0.88 (0.58-1.34)	.6
**Cancer therapy, within 30 d, n (%)**	**n = 1748**	**n = 1000**	**n = 748**		
No active therapy	521 (29.8)	257/521 (49.3)	264/521 (50.7)	Reference	
Active therapy	1227 (70.2)	743/1227 (60.6)	484/1227 (39.4)	0.69 (0.56-0.86)	**<.001**
Conventional chemotherapy	408/1227 (33.3)	243/408 (59.6)	165/408 (40.4)	0.83 (0.63-1.10)	.2
Low-intensity chemotherapy	76/1227 (6.2)	35/76 (46.1)	41/76 (53.9)	0.89 (0.53-1.49)	.7
Molecular-targeted therapy	236/1227 (19.2)	136/236 (57.6)	100/236 (42.4)	0.76 (0.55-1.05)	.1
Immunotherapy	223/1227 (18.2)	158/223 (70.9)	65/223 (29.1)	0.47 (0.33-0.67)	**<.001**
Immunomodulator drugs	116/1227 (9.5)	69/116 (59.5)	47/116 (40.5)	0.67 (0.44-1.03)	.072
Hypomethylating agents	70/1227 (5.7)	35/70 (50.0)	35/70 (50.0)	0.79 (0.47-1.34)	.4
Supportive therapy	47/1227 (3.8)	25/47 (53.2)	22/47 (46.8)	0.64 (0.34-1.20)	.2
Active, not detailed	51/1227 (4.2)	42/51 (82.4)	9/51 (17.6)	0.22 (0.09-0.47)	**<.001**
**Stem cell transplantation, n (%)**	**n = 1727**	**n = 989**	**n = 738**		
No	1431 (82.9)	781/1431 (54.6)	650/1431 (45.4)	Reference	
Yes	296 (17.1)	208/296 (70.3)	88/296 (29.7)	0.79 (0.58-1.06)	.1
Allogenic	98/296 (33.1)	66/98 (67.3)	32/98 (32.7)	1.01 (0.62-1.61)	>.9
Autologous	176/296 (59.5)	127/176 (72.2)	49/176 (27.8)	0.68 (0.46-0.97)	**.039**
CAR T cell	22/296 (7.4)	15/22 (68.2)	7/22 (31.8)	0.96 (0.35-2.39)	>.9
**Vaccination, n (%)**	**n = 1718**	**n = 970**	**n = 748**		
0	1193 (69.4)	559/1193 (46.9)	634/1193 (53.1)	Reference	
1-2	157 (9.1)	113/157 (72.0)	44/157 (28.0)	0.51 (0.34-0.75)	**<.001**
3-4	368 (21.4)	298/368 (81.0)	70/368 (19.0)	0.22 (0.16-0.29)	**<.001**
**COVID-19 therapies, n (%)**	**n = 1781**	**n = 1020**	**n = 761**	Reference: untreated	
Nirmatrelvir/ritonavir	96 (5.4)	93/96 (96.9)	3/96 (3.1)	0.05 (0.01, 0.12)	**<.001**
Remdesivir	249 (14.0)	126/249 (50.6)	123/249 (49.4)	1.59 (1.19, 2.11)	**.002**
Tocilizumab	220 (12.4)	36/220 (16.4)	184/220 (83.6)	10.0 (6.89, 15.0)	**<.001**
Monoclonal antibodies	34 (1.9)	14/34 (41.2)	20/34 (58.8)	2.25 (1.09, 4.80)	**.03**
Corticosteroids	789 (44.3)	213/789 (27.0)	576/789 (73.0)	10.5 (8.32, 13.2)	**<.001**

ALL, acute lymphoid leukemia; AML, acute myeloid leukemia; CAR, chimeric antigen receptor; CI, confidence interval; CLL, chronic lymphocytic leukemia; CML, chronic myeloid leukemia; COVID-19, coronavirus disease 2019; HL, Hodgkin lymphoma; MDS, myelodysplastic syndrome; MPN, myeloproliferative neoplasm; MM, multiple myeloma; n/a, not applicable; NHL, non-Hodgkin lymphoma; OR, odds ratio; SARS-CoV-2, severe acute respiratory syndrome coronavirus-2; y, years. *Adjusted ORs and 95% CIs estimated using multivariable logistic regression models that included sex, age and comorbidities.

Table S2. Baseline characteristics of, and therapy received by, patients with hematologic malignancies according to COVID-19 clinical severity in the Omicron period, and effect of characteristics on odds of having severe/critical disease.

	**Patients with COVID-19 severity data, N = 535**			**Adjusted OR***	
	**All, N = 535 n (%)**	**Mild / Moderate, n = 441 n (% of 'all')**	**Severe / Critical, n = 94 n (% of 'all')**	**OR (95% CI)**	***P*-value**
**Age, y, n (%)**	**n = 534**	**n = 440**	**n = 94**		
<60	175 (32.7)	160/175 (91.4)	15/175 (8.6)	Reference	
60-70	124 (23.2)	101/124 (81.5)	23/124 (18.5)	2.18 (1.08, 4.52)	**.032**
70-80	151 (28.2)	116/151 (76.8)	35/151 (23.2)	2.53 (1.28, 5.19)	**.009**
>80	84 (15.7)	63/84 (75.0)	21/84 (25.0)	2.72 (1.25, 6.06)	**.013**
**Sex, n (%)**	**n = 534**	**n = 440**	**n = 94**		
Male	284 (53.2)	222/284 (78.2)	62/284 (21.8)	Reference	
Female	250 (46.8)	218/250 (87.2)	32/250 (12.8)	0.58 (0.36, 0.93)	**.024**
**Comorbidities, n (%)**	**n = 535**	**n = 441**	**n = 94**		
0	311 (58.1)	271/311 (87.1)	40/311 (12.9)	Reference	
1	143 (26.7)	111/143 (77.6)	32/143 (22.4)	1.37 (0.79, 2.38)	.3
>1	81 (15.1)	59/81 (72.8)	22/81 (27.2)	1.64 (0.84, 3.13)	.1

**Time period (by variant), n (%)**	**n = 535**	**n = 441**	**n = 94**		
Omicron BA.1 or BA.2	321 (60.0)	253/321 (78.8)	68/321 (21.2)	Reference	
Omicron BA.4 or BA.5	214 (40.0)	188/214 (87.9)	26/214 (12.1)	0.46 (0.27, 0.75)	**.003**
**Hematologic malignancy, n (%)**	**n = 534**	**n = 440**	**n = 94**		
Lymphoid malignancy	420 (78.7)	342/420 (81.4)	78/420 (18.6)	Reference (vs myeloid malignancy category)	
NHL	187/420 (44.5)	139/187 (74.3)	48/187 (25.7)	Reference (vs individual malignancies)	
ALL	24/420 (5.7)	22/24 (91.7)	2/24 (8.3)	0.51 (0.08, 2.00)	.4
CLL	60/420 (14.3)	45/60 (75.0)	15/60 (25.0)	0.83 (0.40, 1.64)	.6
HL	22/420 (5.2)	22/22 (100)	0/22 (0)	n/a	>.9
MM	127/420 (30.2)	114/127 (89.8)	13/127 (10.2)	0.30 (0.15, 0.58)	**<.001**
Myeloid malignancy	114 (21.3)	98/114 (86.0)	16/114 (14.0)	0.59 (0.31, 1.05)	.1
AML	51/114 (44.8)	45/51 (88.2)	6/51 (11.8)	0.37 (0.13, 0.89)	**.039**
CML	8/114 (7.0)	6/8 (75.0)	2/8 (25.0)	1.22 (0.17, 5.97)	.8
MDS	27/114 (23.7)	21/27 (77.8)	6/27 (22.2)	0.50 (0.17, 1.32)	.2
MPN	28/114 (24.6)	26/28 (92.9)	2/28 (7.1)	0.18 (0.03, 0.66)	**.026**
**Cancer therapy, within 30 d, n (%)**	**n = 515**	**n = 427**	**n = 88**		
No active therapy	46 (8.9)	38/46 (82.6)	8/46 (17.4)	Reference	
Active therapy	469 (91.1)	389/469 (82.9)	80/469 (17.1)	0.89 (0.41, 2.17)	.8
Conventional chemotherapy	120/469 (25.6)	99/120 (82.5)	21/120 (17.5)	1.08 (0.44, 2.88)	.9
Low-intensity chemotherapy	6/469 (1.3)	4/6 (66.7)	2/6 (33.3)	1.55 (0.18, 10.6)	.7
Molecular-targeted therapy	95/469 (20.3)	74/95 (77.9)	21/95 (22.1)	1.25 (0.50, 3.34)	.6
Immunotherapy	146/469 (31.1)	121/146 (82.9)	25/146 (17.1)	0.88 (0.37, 2.31)	.8
Immunomodulator drugs	39/469 (8.3)	33/39 (84.6)	6/39 (15.4)	0.79 (0.23, 2.59)	.7
Hypomethylating agents	20/469 (4.3)	18/20 (90.0)	2/20 (10.0)	0.36 (0.05, 1.69)	.2
Supportive therapy	10/469 (2.1)	9/10 (90.0)	1/10 (10.0)	0.36 (0.02, 2.52)	.4
Active, not detailed	33/469 (7.0)	31/33 (93.9)	2/33 (6.1)	0.24 (0.03, 1.11)	.1
**Stem cell transplantation, n (%)**	**n = 515**	**n = 427**	**n = 88**		
No	405 (78.6)	332/405 (82.0)	73/405 (18.0)	Reference	
Yes	110 (21.4)	95/110 (86.4)	15/110 (13.6)	1.16 (0.58, 2.23)	.7
Allogenic	36/110 (32.7)	31/36 (86.1)	5/36 (13.9)	1.30 (0.41, 3.48	.6
Autologous	58/110 (52.7)	51/58 (87.9)	7/58 (12.1)	1.02 (0.39, 2.36)	>.9
CAR T cell	16/110 (14.5)	13/16 (81.3)	3/16 (18.8)	1.37 (0.30, 4.71)	.6
**Vaccination, n (%)**	**n = 480**	**n = 396**	**n = 84**		
0	28 (5.8)	24/28 (85.7)	4/28 (14.3)	Reference	
1-2	107 (22.3)	88/107 (82.2)	19/107 (17.8)	1.49 (0.47, 5.76)	.5
3-4	345 (71.9)	284/345 (82.3)	61/345 (17.7)	1.09 (0.38, 3.96)	.9
**COVID-19 therapies, n (%)**	**n = 535**	**n = 441**	**n = 94**	Reference: untreated	
Nirmatrelvir/ritonavir	96 (17.9)	93/96 (96.9)	3/96 (3.1)	0.13 (0.03, 0.35)	**<.001**
Remdesivir	159 (29.7)	103/159 (64.8)	56/159 (35.2)	4.51 (2.80, 7.33)	**<.001**
Tocilizumab	25 (4.7)	3/25 (12.0)	22/25 (88.0)	41.0 (13.5, 179)	**<.001**
Monoclonal antibodies	32 (6.0)	13/32 (40.6)	19/32 (59.4)	7.93 (3.65, 17.9)	**<.001**
Corticosteroids	119 (22.2)	45/119 (37.8)	74/119 (62.2)	33.3 (18.1, 64.5)	**<.001**

ALL, acute lymphoid leukemia; AML, acute myeloid leukemia; CAR, chimeric antigen receptor; CI, confidence interval; CLL, chronic lymphocytic leukemia; CML, chronic myeloid leukemia; COVID-19, coronavirus disease 2019; HL, Hodgkin lymphoma; MDS, myelodysplastic syndrome; MPN, myeloproliferative neoplasm; MM, multiple myeloma; n/a, not applicable; NHL, non-Hodgkin lymphoma; OR, odds ratio; SARS-CoV-2, severe acute respiratory syndrome coronavirus-2; y, years. *Adjusted ORs and 95% CIs estimated using multivariable logistic regression models that included sex, age and comorbidities.

Table S3. Actuarial 30-day and 60-day survival in all patients with hematologic malignancies and COVID-19, overall and in pre-Omicron and Omicron time periods.

	**Overall population**			**Pre-Omicron time-period**			**Omicron time-period**		
	**Survival estimate, % (95% CI)**		***P*-value***	**Survival estimate, % (95% CI)**		***P*-value***	**Survival estimate, % (95% CI)**		***P*-value***
	**30 days**	**60 days**		**30 days**	**60 days**		**30 days**	**60 days**	
**Overall**	78 (76-80)	70 (67-72)		70 (67-73)	60 (56-63)		95 (93-97)	91 (88-94)	
**Age**									
<60 y	93 (91-96)	87 (84-91)		90 (86-94)	82 (77-87)		99 (98-100)	96 (93-100)	
60-70 y	85 (81-89)	77 (72-83)	**<.001**	78 (72-84)	68 (61-76)	**<.001**	98 (96-100)	93 (88-99)	**<.001**
70-80 y	74 (70-78)	64 (59-69)		66 (61-72)	54 (48-60)		92 (88-97)	87 (81-94)	
>80 y	56 (51-62)	48 (42-54)		48 (42-55)	38 (31-45)		86 (78-95)	82 (73-92)	
**Sex**									
Male	76 (74-79)	67 (64-71)	**.04**	69 (66-73)	58 (54-63)	.4	94 (91-97)	89 (85-93)	**.04**
Female	80 (76-83)	73 (69-77)		71 (66-75)	62 (57-67)		97 (94-99)	93 (90-97)	
**Comorbidities**									
0	89 (86-92)	83 (80-87)	**<.001**	81 (77-86)	74 (69-79)	**<.001**	97 (95-99)	94 (90-97)	**<.001**
1	75 (72-79)	65 (61-70)		71 (67-76)	58 (52-63)		90 (85-96)	88 (82-94)	
>1	64 (60-69)	55 (50-61)		57 (52-63)	47 (41-54)		96 (91-100)	86 (77-96)	
**Hematologic malignancy**									
Lymphoid malignancy	78 (76-81)	70 (67-73)		70 (66-73)	59 (55-63)		96 (93-98)	92 (89-95)	
NHL	79 (75-83)	70 (66-75)		71 (65-76)	58 (52-65)		94 (90-98)	89 (84-94)	
ALL	91 (83-100)	72 (57-90)		82 (67-100)	58 (37-90)		100 (100-100)	86 (69-100)	
CLL	71 (65-77)	66 (59-73)		62 (55-70)	55 (46-64)		96 (90-100)	96 (90-100)	
HL	84 (75-93)	81 (72-92)	**.02**	76 (65-90)	73 (60-88)	.3	100 (100-100)	100 (100-100)	.1
MM	79 (75-84)	72 (67-77)		71 (66-77)	61 (55-68)		97 (93-100)	94 (89-99)	
Myeloid malignancy	76 (72-81)	68 (63-73)		71 (66-76)	61 (55-67)		93 (88-99)	88 (80-95)	
AML	75 (67-83)	66 (58-75)		65 (56-76)	55 (45-68)		93 (86-100)	87 (76-98)	
CML	88 (78-100)	88 (78-100)		85 (73-100)	85 (73-100)		100 (100-100)	100 (100-100)	
MDS	65 (57-74)	56 (47-67)		61 (52-71)	51 (41-63)		86 (73-100)	81 (66-100)	
MPN	88 (82-95)	77 (67-87)		85 (77-93)	72 (62-85)		100 (100-100)	92 (79-100)	
**Cancer therapy, within 30 d**									
No active therapy	71 (67-75)	62 (57-67)		68 (63-73)	59 (54-65)		98 (93-100)	89 (79-100)	
Active therapy	80 (78-83)	72 (69-75)		71 (67-74)	59 (55-64)		95 (93-97)	91 (88-94)	
Conventional chemotherapy	74 (70-79)	65 (60-70)		67 (62-74)	56 (49-63)		90 (85-96)	85 (78-92)	
Low-intensity chemotherapy	78 (68-89)	63 (50-79)		77 (67-89)	62 (48-81)		83 (58-100)	67 (38-100)	
Molecular-targeted therapy	82 (77-88)	76 (70-83)	**.001**	73 (65-81)	65 (56-75)	>.9	96 (91-100)	92 (85-99)	.8
Immunotherapy	90 (85-94)	84 (78-89)		74 (64-86)	60 (48-74)		98 (95-100)	96 (92-99)	
Immunomodulator drugs	78 (70-87)	72 (63-82)		71 (60-83)	61 (49-76)		92 (83-100)	92 (83-100)	
Hypomethylating agents	74 (64-87)	71 (60-85)		66 (53-83)	62 (48-80)		94 (84-100)	94 (84-100)	
Supportive therapy	70 (56-87)	55 (40-76)		65 (50-84)	49 (33-72)		100 (100-100)	100 (100-100)	
Active, not detailed	97 (93-100)	90 (80-100)		93 (80-100)	85 (68-100)		100 (100-100)	93 (82-100)	
**Stem cell transplantation**									
No	75 (73-78)	67 (64-70)		67 (64-70)	57 (53-61)	**<.001**	94 (92-97)	91 (88-94)	.5
Yes	91 (88-95)	83 (78-89)	**<.001**	86 (81-92)	76 (69-84)		99 (97-100)	93 (87-99)	
Allogenic	93 (88-99)	80 (71-90)		88 (80-98)	74 (62-89)	.5	100 (100-100)	88 (76-100)	.7
Autologous	90 (85-95)	86 (80-92)		85 (78-93)	80 (72-90)	**<.001**	98 (95-100)	95 (89-100)	.3
**Vaccinations**									
0	70 (67-73)	59 (55-62)		69 (66-72)	58 (54-61)		96 (88-100)	96 (88-100)	
1-2	92 (87-96)	87 (81-93)	**<.001**	84 (74-95)	76 (64-89)	**<.001**	96 (92-100)	93 (88-99)	.2
3-4	94 (92-97)	90 (86-94)		91 (80-100)	91 (80-100)		94 (92-97)	90 (86-94)	
**COVID-19 therapies**									
**Nirmatrelvir/ritonavir**									
No	77 (74-79)	68 (66-71)	**<.001**	NE (NE-NE)	NE (NE-NE)	n/a	94 (92-97)	90 (87-93)	.2
Yes	99 (96-100)	96 (91-100)		NE (NE-NE)	NE (NE-NE)		99 (96-100)	96 (91-100)	
**Remdesivir**									
No	76 (73-78)	69 (66-71)	**<.03**	69 (66-72)	59 (56-63)	.1	95 (93-98)	94 (91-97)	**<.001**
Yes	90 (86-94)	76 (70-83)		82 (73-91)	65 (54-77)		95 (91-99)	83 (76-91)	
**Tocilizumab**									
No	79 (77-82)	72 (69-74)	**<.001**	71 (68-74)	61 (57-65)	.3	96 (94-98)	92 (89-95)	**<.001**

Yes	69 (63-76)	58 (51-65)		68 (61-75)	55 (48-64)		78 (63-97)	73 (57-94)	
**Monoclonal antibodies**									
No	78 (75-80)	69 (67-72)	.1	NE (NE-NE)	NE (NE-NE)	n/a	95 (93-97)	92 (89-95)	.3
Yes	93 (83-100)	80 (65-97)		NE (NE-NE)	NE (NE-NE)		93 (83-100)	80 (65-97)	
**Corticosteroids**									
No	89 (87-91)	85 (82-87)	**<.001**	82 (78-85)	76 (71-80)	**<.001**	98 (97-99)	95 (92-97)	**<.001**
Yes	65 (61-69)	53 (49-57)		62 (58-66)	48 (44-53)		85 (78-92)	77 (69-86)	

ALL, acute lymphoid leukemia; AML, acute myeloid leukemia; CAR, chimeric antigen receptor; CI, confidence interval; CLL, chronic lymphocytic leukemia; CML, chronic myeloid leukemia; COVID-19, coronavirus disease 2019; HL, Hodgkin lymphoma; IQR, interquartile range; MDS, myelodysplastic syndrome; MPN, myeloproliferative neoplasm; MM, multiple myeloma; n/a, not applicable; NE, not estimable; NHL, non-Hodgkin lymphoma; SARS-CoV-2, severe acute respiratory syndrome coronavirus-2; y, years. *P-values estimated using log-rank test.
